# Component Identification of Phenolic Acids in Cell Suspension Cultures of *Saussurea* *involucrata* and Its Mechanism of Anti-Hepatoma Revealed by TMT Quantitative Proteomics

**DOI:** 10.3390/foods10102466

**Published:** 2021-10-15

**Authors:** Junpeng Gao, Yi Wang, Bo Lyu, Jian Chen, Guang Chen

**Affiliations:** 1 College of Life Science, Jilin Agricultural University, Changchun 130118, China; joepgao@163.com; 2 College of Food Science and Engineering, Jilin Agricultural University, Changchun 130118, China; Wangyi284419@163.com (Y.W.); michael_lvbo@163.com (B.L.); cj15068528679@163.com (J.C.); 3 College of Food Science, Northeast Agricultural University, Harbin 150030, China

**Keywords:** *S. involucrata*, anti-hepatoma, traditional Chinese medicine, pharmacological mechanism, proteomics

## Abstract

*Saussurea involucrata* (*S. involucrata*) had been reported to have anti-hepatoma function. However, the mechanism is complex and unclear. To evaluate the anti-hepatoma mechanism of *S. involucrata* comprehensively and make a theoretical basis for the mechanical verification of later research, we carried out this work. In this study, the total phenolic acids from *S. involucrata* determined by a cell suspension culture (ESPI) was mainly composed of 4,5-dicaffeoylquinic acid, according to the LC-MS analysis. BALB/c nude female mice were injected with HepG2 cells to establish an animal model of liver tumor before being divided into a control group, a low-dose group, a middle-dose group, a high-dose group, and a DDP group. Subsequently, EPSI was used as the intervention drug for mice. Biochemical indicators and differences in protein expression determined by TMT quantitative proteomics were used to resolve the mechanism after the low- (100 mg/kg), middle- (200 mg/kg), and high-dose (400 mg/kg) interventions for 24 days. The results showed that EPSI can not only limit the growth of HepG2 cells in vitro, but also can inhibit liver tumors significantly with no toxicity at high doses in vivo. Proteomics analysis revealed that the upregulated differentially expressed proteins (DE proteins) in the high-dose group were over three times that in the control group. ESPI affected the pathways significantly associated with the protein metabolic process, metabolic process, catalytic activity, hydrolase activity, proteolysis, endopeptidase activity, serine-type endopeptidase activity, etc. The treatment group showed significant differences in the pathways associated with the renin-angiotensin system, hematopoietic cell lineage, etc. In conclusion, ESPI has a significant anti-hepatoma effect and the potential mechanism was revealed.

## 1. Introduction

*Saussurea involucrata* (*S. involucrata*) is a rare and slow-growing herb growing in the Tianshan and Altay Mountains of Xinjiang Province, China, at altitudes over 2600 m. *S. involucrata* has the effect of promoting blood circulation, relaxing tendons, dispersing cold, and removing dampness [1]. It is mainly used for the treatment of coughs, rheumatoid arthritis, high-altitude response, and stomach pain [2].

According to previous studies, *S. involucrata* has many bioactive compounds, including lignans [3], flavonoids [3], coumarins [4], sesquiterpene lactones [5], steroids, and phenylpropanoids [6]. These compounds show a wide range of biological activity, including anti-inflammatory [7], anti-aging [8], antioxidant, anti-fatigue [8], and anti-tumor effects [2]. In addition, it has been shown that the methanol extracted from *S. involucrata* could inhibit the expression of cytokines stimulated by lipopolysaccharide (LPS), and the ethyl acetate extract could effectively inhibit phosphorylation and activation of the EGFR, Akt, and STAT3 pathway in PC-3 cells [9]. Meanwhile, *S. involucrata* also showed strong anti-rheumatic activity in the clinical environment [10]. It can be seen that *S. involucrata* has an obvious inhibitory effect on some inflammation.

Due to the unique growth environment and the long growth cycle of *S. involucrata*, artificial cultivation is very difficult. In addition, with the market demand and overexploitation, the stock of many rare medicinal plants had decreased sharply. Using biotechnology to regulate plants’ secondary metabolism, obtain useful secondary metabolites and clarify their biosynthetic pathway has become an important goal of researchers [11]. Through the identification of metabolites and enzymes in the process of biosynthesis, people can better understand the metabolic pathway of plant components, combined with genomic and proteomic analysis technologies [12]. Under artificial control, a large number of products can be obtained in a short time from cell suspension cultures of *S. involucrata*, which can ensure stable product quality and high-efficacy components, and realize large-scale industrial production, to solve the problem of a shortage of *S. involucrata*.

This study was based on the previous research results of our team. We prepared the total phenolic acids from *S. involucrata* through a cell suspension culture (EPSI). However, its mechanism of action was not clear enough to guide clinical applications [13]. In order to explore the potential anti-hepatoma mechanism of EPSI more comprehensively and make a theoretical basis for later verification, this study depended on tumor-bearing BALB/c mice model combined with proteomics technology to verify the mechanism of action of *S. involucrata* on human hepatoma, while realizing the modern utilization of *S. involucrata* resources and the accurate analysis of its drug theory.

## 2. Materials and Methods

### 2.1. Cell Suspension Culture of S. involucrata

A cell suspension culture of *S. involucrata* was provided by the Engineering Research Center of Bioreactor and Pharmaceutical Development, Ministry of Education (College of Life Science, Jilin Agricultural University).

### 2.2. Extraction of the Phenolic Acids in Cell Suspension Cultures of S. involucrata (EPSI)

Phenolic acids are soluble in organic solvents, so the total phenolic acids were extracted by the organic solvent extraction method [14]. The method is as follows: The cell suspension culture of *S. involucrata* was mixed 1:8 (m/v) with absolute alcohol, heated at 50 °C, and stirred for 15 min. After the supernatant was collected, 8× absolute alcohol was added to the precipitate and stirred for 2 h; the above steps were repeated twice. The collected supernatant was filtered and subjected to rotary evaporation at 60 °C. After the rotary distillation, the raw materials were collected with water, and the organic phase was extracted with ethyl acetate. The above steps were repeated 3 times. After the materials had been subjected to rotary evaporation at 60 °C, the concentrate (EPSI) was collected and freeze-dried at −80 °C for storage.

### 2.3. Component Identification by Liquid Chromatography-Mass Spectrometry (LC-MS)

The composition of EPSI was analyzed by LC-MS (Thermo, Ultimate 3000LC, Q Exactive HF) [15,16,17]. The conditions were as follows. Chromatographic column: C18 column (Zorbax Eclipse C18 [1.8 μm × 2.1 × 100 mm]); separation conditions: column temperature: 30 °C; current speed: 0.3 mL/min; Mobile Phase A: water + 0.1% formic acid (CAS: 64-18-6, Xiya Chemical Technology (Shandong) Co., Ltd., Linyi, China), Mobile Phase B: acetonitrile (CAS: 75-05-8, Merck KGaA, Darmstadt, Germany); injection volume: 2 μL; autosampler temperature: 4 °C. The process of gradient elution is shown in Table 1.

The positive mode was used for analysis: Heater temperature: 325 °C; sheath gas velocity: 45 arb; flow rate of the auxiliary gas: 15 arb; purge gas flow rate: 1 arb; electrospray voltage: 3.5 kV; capillary temperature: 330 °C; S-lens RF level: 55%; scanning mode: full scan (M/Z 100–1500) and data dependent secondary mass spectrometry (dd-ms2, TopN = 10); resolution: 120,000 (primary MS) and 60,000 (secondary MS); collision mode: high-energy collision dissociation (HCD).

Compound Discoverer 3.1 Software was used for correction of the retention time, peak identification, and peak extraction. According to the secondary MS spectrum information, the substances were identified by using the Thermo mzCloud Online Database (https://www.mzcloud.org/, accessed on 23 August 2021).

### 2.4. Effect of EPSI on the Multiplication of HepG2 Cells In Vitro

Hepatic cellular carcinoma (HCC) cell lines HepG2 were purchased from Bluef Biotechnology Development Co. Ltd. (Shanghai, China) and cultured in Dulbecco’s Modified Eagle Medium (DMEM) (Thermo Fisher Scientific, Waltham, MA, USA) supplemented with 10% fetal bovine serum (FBS) (Thermo Fisher Scientific, Waltham, MA, USA).

After resuscitation, cells at the logarithmic phase were taken for standby. We adjusted the cell concentration to 10^4^ units/mL and added them to a 96-well culture plate at a concentration of 100 μL/well for culturing (37 °C, CO_2_ concentration: 5%, 24 h). The old medium was discarded after culturing, and a blank group with 100 μL of 5% medium was added. For the experimental group, EPSI samples of different concentrations were added and then cultured at 37 °C and 5% CO_2_ for 72 h.

CCK8 (CCK-8 Cell Proliferation and Cytotoxicity Assay Kit, Solarbio, Beijing, China) and MTT (MTT Cell Proliferation and Cytotoxicity Assay Kit, Solarbio, Beijing, China) were used to evaluate the inhibitory effect of EPSI on HepG2 cells in vitro. All operations were carried out according to the operating instructions.

### 2.5. Acute Toxic Test

A fixed-dose procedure (FDP) was used to verify the acute toxicity of EPSI [18,19]. The dosage was set at 2000 mg/kg. Six- to eight-week-old BALB/c nude female mice (Beijing Vital River Laboratory Animal Technology Co. Ltd., Beijing, China) were used as the test animal. Intraperitoneal injection (I.P.) was used as the mode of administration. It was considered that EPSI had no acute toxicity if the LD50 was >2000 mg/kg.

### 2.6. Establishment of the Animal Model

Six- to eight-week-old BALB/c nude female mice were purchased from Beijing Vital River Laboratory Animal Technology Co. Ltd. (Beijing, China). All mice were housed under controlled conditions in individual cages at 22 ± 3°C and 60–70% relative humidity with a 12 h dark/light cycle in a specific germ-free environment and were allowed free access to sterile food and water. After 1 week of acclimation, each animal was subcutaneously injected with 10^6^ units of HepG2 cells. When there were measurable tumors with an equivalent volume, the experiment animals were randomly divided into five groups. The groups consisted of the control group (no gavage, free feeding, n = 8), the low-dose group (100 mg/kg EPSI gavage for 24 days, free feeding, n = 8), the middle-dose group (200 mg/kg EPSI gavage for 24 days, free feeding, n = 8), the high-dose group (400 mg/kg EPSI gavage for 24 days, free feeding, n = 8) and the DDP (PtCl_2_[NH_3_]_2_) group (10% DDP intraperitoneal injection for 10 days, free feeding, n = 8). All operations were performed in a sterile environment.

The weights and the tumor volumes of the experimental animals in each group were measured once every 2 days at night during the intervention. The experimental animals were sacrificed when there were significant differences in tumor volume. The tumors were removed and stored at −80 °C after eyeball blood collection. All animal experiments were approved by the Laboratory Animal Welfare and Ethics Committee of Jilin Agricultural University (No. 20190410005), following National Research Council Guidelines.

### 2.7. Hematoxylin and Eosin (H&E) Staining

The tumor tissues obtained as described in Part 2.4 were separated by about 0.2 mm^3^, without being frozen, and were fixed in a 4% paraformaldehyde solution for 12 h at room temperature and embedded in paraffin. After cutting the paraffin into 5 µm sections, the slides were dewaxed, rehydrated, and stained with 1% H&E at room temperature as described previously.

### 2.8. Enzyme Linked Immunosorbent Assay (ELISA) analysis

Blood was drawn from the eyeball and centrifuged (3000× *g*, 10 min, 4 °C) to obtain the serum. Tumor necrosis factor-α (TNF-α) was measured according to an ELISA kit protocol (No. ML002095, Shanghai Enzyme-linked Biotechnology Co., Ltd., Shanghai, China).

### 2.9. Tandem Mass Tag (TMT) Quantitative Proteomics

The process of TMT quantitative proteomics was basically consistent with the research methods for animal tissues in other studies [20]. The process is briefly described as follows [21,22,23].

#### 2.9.1. Total Protein Extraction 

The sample was ground into powder at a low temperature and transferred to a tube (cooled by liquid nitrogen). A certain amount of a PASP lysis buffer (100 mM NH_4_HCO_3_, 8 M Urea, pH = 8), was added, mixed, and ultrasonicated for 5 min under an ice bath to full splitting. The product was centrifuged at 4 °C and 12,000× *g* for 15 min. Next, 10 mM DTT (DL-dithiothreitol) was added to the supernatant and reacted at 56 °C for 1 h. Sufficient IAM (iodoacetamide) was then added and reacted at room temperature without light for 1 h. After completing the above process, 4× the volume of acetone (precooled at 20 °C) was added, precipitated at −20 °C for 2 h, and centrifuged at 4 °C and 12,000× *g* for 15 min, and the precipitate was collected. After that, 1 mL of acetone (precooled at 20 °C) was added to resuspend the precipitate, then the mixture was centrifuged at 4 °C and 12,000× *g* for 15 min, the precipitate was collected, air-dried, and an appropriate amount of a protein solution dissolution buffer (8 M urea, 100 mM TEAB (triethylammonium bicarbonate); pH 8.5) was added to dissolve the protein precipitate.

#### 2.9.2. Protein Quality Test

The Bradford protein quantitative kit was used to prepare a BSA (bovine serum albumin) standard protein solution according to the instructions, and the concentration gradient range was from 0 to 0.5 g/L. The BSA standard protein solution with different concentration gradients and the sample solution were taken to be tested at different dilution ratios and were added into a 96-well plate, the volume was made up to 20 µL, and each gradient was repeated 3 times. After that, 180 µL of a G250 staining solution was added immediately, and the mixture was placed at room temperature for 5 min, and the absorbance at 595 nm was measured. The standard curve was drawn with the absorbance of the standard protein solution, and we calculated the protein concentration of the sample to be tested. For gel electrophoresis, 20 μg of the protein sample was loaded to 12% SDS-PAGE (Sodium dodecyl sulfate—Polyacrylamide gel electrophoresis). The electrophoresis conditions were 80 V for 20 min in concentrated gel, then 120 V for 90 min in separation gel. After electrophoresis, Coomassie brilliant blue R-250 staining was performed to decolorize the sample until the bands were clear.

#### 2.9.3. TMT Labeling of Peptides

A protein sample was taken and a DB dissolution buffer (8 M urea, 100 mM TEAB; pH 8.5) was added to make up the volume to 100 μL. Trypsin and the 100 mM TEAB buffer were added, mixed well, and digested at 37 °C for 4 h, then trypsin and CaCl_2_ were added overnight. Formic acid was added to adjust the pH to be less than 3, then the sample was mixed well and centrifuged at room temperature at 12,000× *g* for 5 min. The supernatant was passed through the C18 demineralizer column slowly, then washed 3 times with a washing buffer (0.1% formic acid, 3% acetonitrile). After that, an appropriate amount of an elution buffer (0.1% formic acid, 70% acetonitrile) was added to collect the filtrate and freeze-dried. The lyophilized sample was reconstituted by adding 100 μL of 0.1 M TEAB buffer and 41 µL of TMT labeling reagent dissolved in acetonitrile. The reaction was reversed and mixed at room temperature for 2 h. After that, 8% ammonia was used to stop the reaction, and the samples marked with equal volume were mixed, desalted, and lyophilized [24].

#### 2.9.4. Separation of Fractions

The mobile phase composition was as follows: Mobile Phase A: 2% acetonitrile, with the pH adjusted to 10.0 using ammonium hydroxide; B: 98% acetonitrile. The lyophilized powder was dissolved with Solution A and centrifuged at 12,000× *g* at room temperature for 10 min. The L-3000 HPLC (high-performance liquid chromatography) system with a C18 column (Waters BEH C18, 4.6 × 250 mm, 5 μm; column temperature: 45 ° C) was used to test the sample. The specific elution gradient is shown in Table 1. One tube was collected every minute and divided into 10 fractions. After freeze-drying, 0.1% formic acid was added to dissolve each fraction. The details of the elution gradient are shown in Table 2. One tube was collected per minute and divided into 10 fractions. After freeze-drying, 0.1% formic acid (FA) was added to dissolve each fraction.

#### 2.9.5. LC-MS/MS Analysis

The mobile phase composition was as follows: Mobile Phase A: 0.1% formic acid; Mobile Phase B: 80% acetonitrile and 0.1% formic acid. Next, 1 μg of the supernatant of each fraction was injected and tested. The EASY-nLC 1200 UHPLC system (Thermo Fisher) was coupled with the C18 Nano-Trap column (4.5 cm × 75 μm, 3 μm) as a homemade analytical column and the C18 Nano-Trap column (15 cm × 150 μm, 1.9 μm) as a homemade analytical column. The linear elution gradient is shown in Table 3. A Q Exactive HF-X mass spectrometer (Thermo Fisher) was used with the ion source of Nanospray Flex (ESI) to analyze the samples. The conditions were as follows: spray voltage: 2.1 kV; ion transport capillary temperature: 320 °C, full scan range: *m/z* 350 to 1500; primary MS resolution: 60,000 (at *m/z* 200); automatic gain control (AGC) target value: 3 × 10^6^; maximum ion injection time: 20 ms. The top 40 precursors with the highest abundance in the full scan were selected and fragmented by higher-energy collisional dissociation (HCD) and analyzed by MS/MS. The conditions were as follows: resolution: 30,000 (at *m/z* 200); AGC target value: 5 × 10^4^; maximum ion injection time: 54 ms; normalized collision energy: 32%; intensity threshold: 1.2 × 10^5^; dynamic exclusion parameter: 20 s.

#### 2.9.6. Data Analysis

##### Identification and Quantitation of Protein

The resulting spectra from each run were searched separately against the homo_sapiens_uniprot_2021_3_9 (194,557 sequences) database by the search engine Proteome Discoverer 2.4 (PD 2.4, Thermo). The search parameters were set as follows: mass tolerance for the precursor ion was 10 ppm and the mass tolerance for production was 0.02 Da. Carbamidomethyl was specified as a fixed modification; oxidation of methionine (M), and TMT plex were specified as dynamic modifications. Acetylation, TMT plex, Met loss, and Met-loss + Acetyl were specified as *N*-terminal modifications in PD 2.4. A maximum of 2 missed cleavage sites was allowed.

The software package PD 2.4 was used to improve the quality of the results for further filtering. Peptide spectrum matches (PSMs) with a reliability of more than 99% were identified as PSMs, which had to contain 1 unique peptide (5 unique peptides) or more. The identified PSMs and proteins with a FDR no more than 1.0% were retained and tested. The protein quantitation results were statistically analyzed by T-tests. The proteins whose quantitation was significantly different between the high-dose group and the control group (*p* < 0.05 and |log_2_FC| > 0.25 (FC > 1.2 or FC < 0.83) (fold change, FC)) were defined as differentially expressed proteins (DEP).

##### Functional Analysis of Protein and DEP

Gene Ontology (GO) and InterPro (IPR) functional analyses were conducted using the interproscan program against the non-redundant protein database (including Pfam, PRINTS, ProDom, SMART, ProSite, and PANTHER) [21], and the COG (Clusters of Orthologous Groups) and KEGG (Kyoto Encyclopedia of Genes and Genomes) databases were used to analyze the protein families and pathways. DEPs were used for volcanic map analysis, cluster heat map analysis, and GO, IPR, and KEGG enrichment analysis [22]. The probable protein–protein interactions were predicted using the STRING-db server (http://string.embl.de/, accessed on 23 August 2021) [23].

### 2.10. Statistical Analysis

All the data are presented as means ± standard deviation (SD). Statistical analysis was carried out by GraphPad Prism version 6 (GraphPad Software, Inc., San Diego, CA, USA). Difference comparisons were carried out using the T-test or one-way analysis of variance (ANOVA) using IBM SPSS 25.0 (SPSS Inc., Chicago, CA, USA); *p* < 0.05 was considered statistically significant.

## 3. Results and Discussion

### 3.1. Composition of the Extract of the Phenolic Acids in Cell Suspension Cultures from S. involucrata (EPSI)

**Figure 1:**
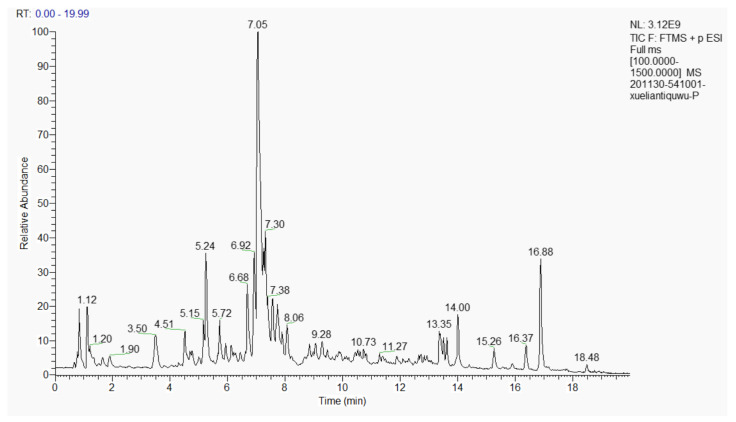
Total ion current (TIC) of EPSI.

The total ion current is shown in Figure 1. The information of the qualitative metabolite results (positive mode) based on Figure 1 are shown in Table 4. In EPSI, 13 substances with a content of more than 1% were detected, including 4,5-dicaffeoylquinic acid (29.000%), linolenyl alcohol (8.453%), 7-hydroxycoumarine (7.691%), chlorogenic acid (6.134%), metronidazole (3.523%), apigenin 7-(6″-crotonylglucoside) (2.814%), 9-oxo-10(E),12(E)-octadecadienoic acid (2.618%), cynaroside (2.382%), phthalic anhydride (1.956%), hexadecanamide (1.883%), 3,4,5-tricaffeoylquinic acid (1.773%), diisobutyl phthalate (1.139%), and triethyl phosphate (1.061%). Many of them were reported to have biological activity and even pharmacology.

4,5-Dicaffeoylquinic acid (isochlorogenic acid C), as the largest substance in the extract, is considered to have the function of promoting blood circulation in traditional Chinese medicine [2]. It has now been proven that it can improve the function of islet cells [25] and even inhibit cancer [26,27]. There are reasons to believe that it is the main anti-cancer ingredient of *S. involucrata*. As a lipid compound, linolenyl alcohol has been rarely reported as a functional compound; only a few reports showed that it has a certain antiviral activity [28,29]. 7-Hydroxycoumarine also occupied a certain ratio in the extract. 7-Hydroxycoumarin is one of the main chemical constituents of *Angelica dahurica* and can inhibit the growth of malignant tumors [30]. Meanwhile, its derivatives also have typical anti-inflammatory activities [31,32]. Chlorogenic acid is one of the main antibacterial and antiviral components of honeysuckle, which has good antioxidant and antibacterial properties [33,34]. 9-Oxo-10(E),12(E)-octadecadienoic acid is a potent PPARα agonist that decreases triglyceride accumulation [35], and can also induce the apoptosis of cancer cells [36].

Thus, after the identification of the components of EPSI, a variety of components with pharmacological effects were identified. These proved that EPSI may have potential anti-hepatoma ability.

### 3.2. Growth Inhibition of EPSI in HepG2 Cells

**Figure 2:**
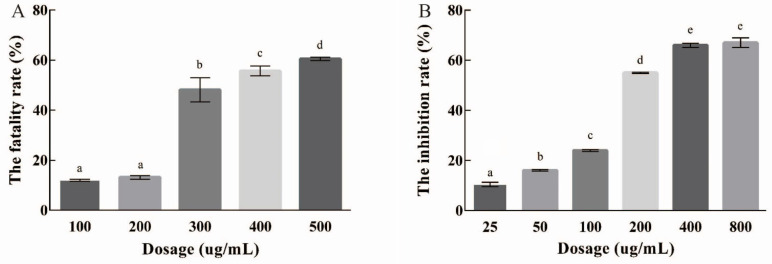
The growth inhibition of EPSI in HepG2 cells: (**A**) CCK8; (**B**) MTT. Different lowercase letters represent significant differences (*p* < 0.05).

The CCK8 and MTT methods were used to determine EPSI’s inhibition of HepG2 cell proliferation. As shown in Figure 2A (CCK8), the inhibitory ability of EPSI on the proliferation of HepG2 was at a low level (< 20%) when the concentration was 100–200 μg/mL. When the dose concentration was increased to 300 μg/mL, the proliferation inhibition of EPSI in HepG2 cells increased to about 50%, which was significantly different from that at a low dose (*p* < 0.05). When the dosage was over 400 μg/mL, the inhibitory ability of EPSI in HepG2 increased slightly, and the maximum rate was about 58%. This result showed that the IC50 value of EPSI was between 100–200 μg/mL for the CCK8 method. When the dosage was between 100–300 μg/mL, EPSI showed an obvious inhibition ability in HepG2 cells.

In Figure 2B, we measured the growth inhibition of EPSI in HepG2 cells by the MTT method. When the dose concentration was 25–100 μg/mL, the proliferation of HepG2 cells was still at a high level and the maximum inhibition was less than 25%. When the dosage was 200 μg/mL, EPSI increased the inhibition of HepG2 cells significantly (55%, *p* < 0.05). When the dosage was 400 μg/mL or higher, the inhibition ability of HepG2 improved slightly. The maximum inhibition ability of EPSI in HepG2 cells was about 67%. These results showed that the IC50 value of EPSI was 100–200 μg/mL for the MTT method. When the dosage was 100–300 μg/mL, EPSI showed good inhibition in vitro.

### 3.3. Acute Toxicity Test

The fixed-dose procedure (FDP), a classic way to determine the acute toxicity, was used to verify the acute toxicity of EPSI. The acute toxicity test of EPSI in BALB/c mice showed a LD50 of >2 g/kg, indicating that the product was safe for the experiment of the next stage. This toxicity was lower than that of conventional drugs with anticancer activity [37,38,39].

### 3.4. Effect of EPSI on Tumors in BALB/c Nude Mice

**Figure 3:**
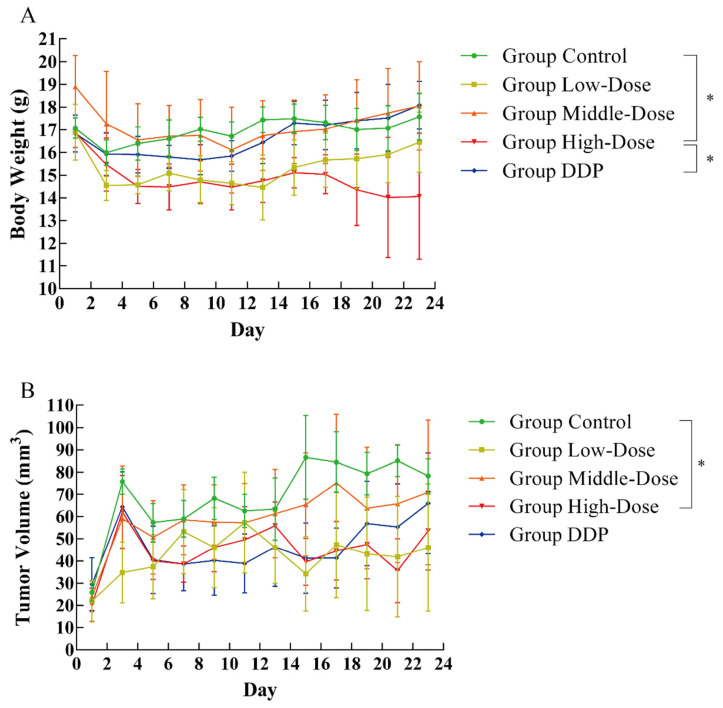
Bodyweight and tumor volume changes in the animal experiment during treatment. (**A**) Bodyweight changes and (**B**) tumor volume changes (n = 8; *: *p* < 0.05) at Day 23.

The bodyweight changes of all experimental animals are shown in Figure 3A. Compared with the control group, the weight of the animals in the high-dose group showed a significant reduction (*p* < 0.05), which happened from Day 15. During the use of anticancer drugs, the situation of weight loss occurs [40,41]. The intersection of liver cancer, anticancer drugs, and weight change may be the thyroid gland [42,43], which is closely related to the proliferation of liver cancer cells and changes in bodyweight. Whether EPSI affects the thyroid needs further study. However, for cancer patients, keeping correct weight is necessary to maintain efficacy [44,45].

**Figure 4:**
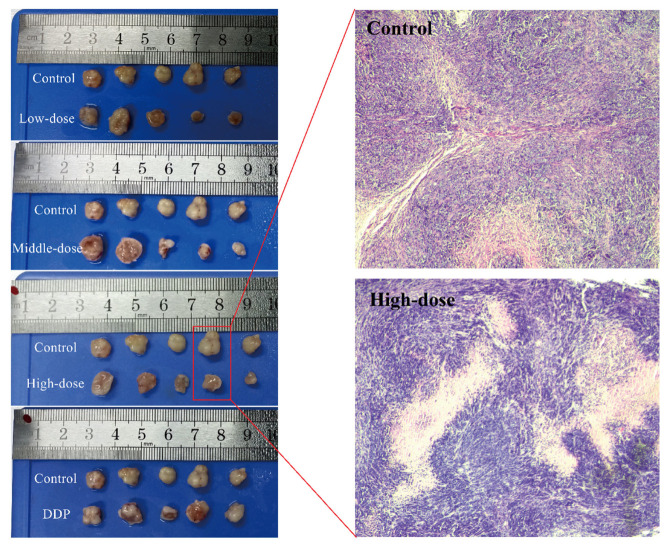
The state and hematoxylin and eosin (H&E) staining of tumors at the sacrifice of experimental animals (n = 8, representative samples are shown). Low dose: 100 mg/kg EPSI gavage for 24 days; middle dose: 200 mg/kg EPSI gavage for 24 days; high dose: 400 mg/kg EPSI gavage for 24 days; DDP: 10% DDP intraperitoneal injection for 10 days.

The changes in tumor volume are shown in Figure 3B. The state of the tumor at the sacrifice of experimental animals is shown in Figure 4. Because of the individual differences of experimental animals, the tumor volume was not very consistent. However, EPSI can certainly inhibit the growth of tumors, because the tumor volume of the treatment group (low/middle/high-dose groups) was lower than that of the control group (Figure 3B). A significant difference was shown between the high-dose group and the control group (*p* < 0.05). Meanwhile, the tumor volume of the medium-dose group and the high-dose group was significantly smaller than that of the DDP group (*p* < 0.05), showing good tumor inhibitory activity (Figure 3B). Observation of the tumor state after animal sacrificed also revealed the same situation (Figure 4): the tumor state of the treatment group was significantly different from that of other groups.

The results of H&E staining showed the state of the tumor tissue directly, and the results of the control group and the high-dose group are shown in Figure 4. Compared with the control group, the tumors in the high-dose group showed the significant reduction and destruction of tumor tissue. The tumor tissue in the control group was dense and uniform, while that of the high-dose group was disordered and less density, with even a lack of tissue. This result showed that EPSI had a significant inhibitory effect on hepatoma tumors, especially at high doses. Because EPSI passed the acute toxicity test, the high-dose group and the control group were selected for quantitative proteomic analysis.

**Figure 5:**
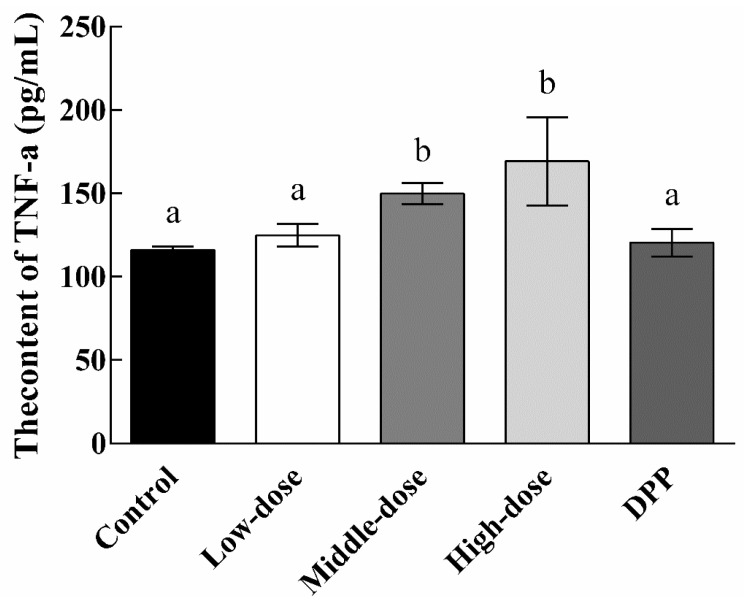
TNF-α content in the serum of experimental animals (n = 8). Low dose: 100 mg/kg EPSI gavage for 24 days; middle dose: 200 mg/kg EPSI gavage for 24 days; high dose: 400 mg/kg EPSI gavage for 24 days; DDP: 10% DDP intraperitoneal injection for 10 days. Different lowercase letters represent significant differences (*p* < 0.05).

The effects of EPSI intake on TNF-α are shown in Figure 5. As a tumor necrosis factor, TNF-α is an important serological indicator used to measure the severity of a tumor. As shown in Figure 5, the serum TNF-α level in the control group was the lowest (116.11 pg/mL), which was basically the same as that in the DDP group (120.42 pg/mL). The level of TNF-α in the treatment group showed an obvious upward trend with an increase in the dosage. Although the low-dose group (124.82 pg/mL) increased slightly, no significant differences (*p* > 0.05) were seen compared with the control group. TNF-α in the middle-dose group (149.89 pg/mL) and the high-dose group (169.29 pg/mL) were significantly increased (*p* < 0.05) compared with the control group.

BALB/c mice are innately immune-deficient animals. The test animals have no thymus, resulting in T-cell defects and dysfunction. At this time, we can judge that the higher the TNF-α content, the stronger the immune response of the tested animals and the more obvious the improvement of their immunity. Therefore, these results mean that with the increase in the EPSI dosage, the limited immune system of the test animals was activated and produced a stronger immune response in the case of a lack of immune function.

### 3.5. Protein Expression Differences Induced by EPSI

**Figure 6:**
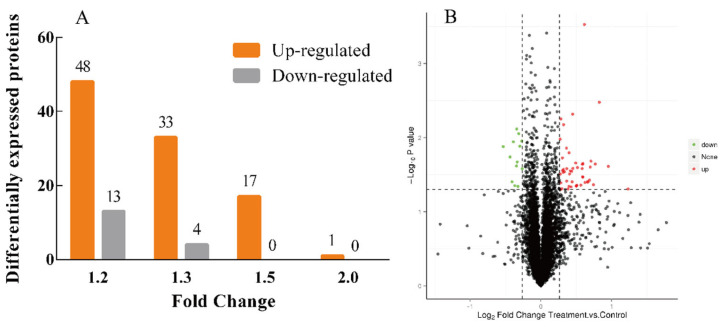
Distribution of proteins identified in tumor of BALB/c mice (n = 3). (**A**) Up-regulated and down-regulated of the DE proteins (Filtered with the threshold value of expression fold change, *p* < 0.05); (**B**) Volcano plot of the DE proteins. The X-axis was the fold change, and the Y-axis was the significant difference *p* value. Red dots represented the expression of up-regulated and green dots represented the down-regulated.

The proteomic data of the tumors from the high-dose group of BALB/c mice are shown in Figure 6A. In total, 116 differentially expressed proteins (DE proteins) and 6787 proteins were identified in this experiment. On the whole, the number of upregulated DE proteins (99) was far more than that of downregulated DE proteins (17) between the control group and high-dose group after the intervention, and the upregulated DE protein amounts in the high-dose group were over 3 times higher than that in the control group when the fold change was 1.2×. The volcano map of the DE proteins with a 1.2× fold change is shown in Figure 6B. According to Figure 6B, the number of upregulated and downregulated proteins in the treatment group had unique proteins.

### 3.6. GO Enrichment of Differentially Quantified Proteins

**Figure 7:**
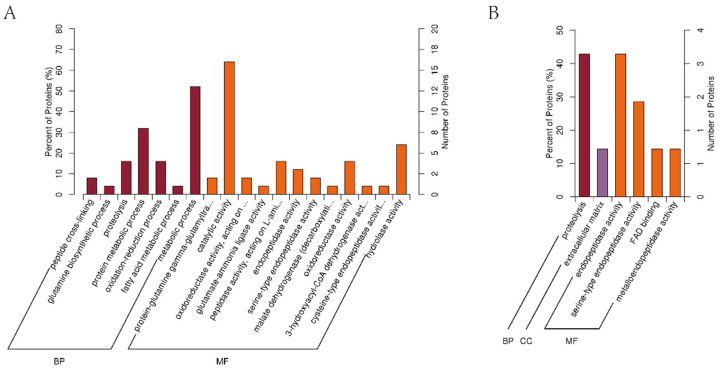
GO enrichment results. (CC: cell component; MF: molecular function; BP: biological process; n = 3). (**A**) Upregulated DE proteins (*p* < 0.05); (**B**) downregulated DE proteins (*p* < 0.05).

Following the GO classifications for biological process (BP), cellular component (CC), and molecular function (MF), the enrichment results were examined to compare the functional correlations of DE proteins; the results are shown in Figure 7. In the upregulated proteins (Figure 7A), the proteins had functions in biological process and molecular function, but not cell component. These proteins are mainly concentrated (>20%) in protein metabolic processes (BP), metabolic processes (BP), catalytic activity (MF), and hydrolase activity (MF). In the downregulated proteins (Figure 7B), by comparison, all three classifications of proteins existed, and their functions showed a high concentration. Proteolysis (BP), endopeptidase activity (MF), and serine-type endopeptidase activity (MF) were the main roles of these proteins. These results suggest that the intake of EPSI associated with the anti-hepatoma effect may be achieved through specific mechanisms.

### 3.7. Specific Regulation Pathways for Inhibiting Liver Tumor Proliferation by EPSI

**Figure 8:**
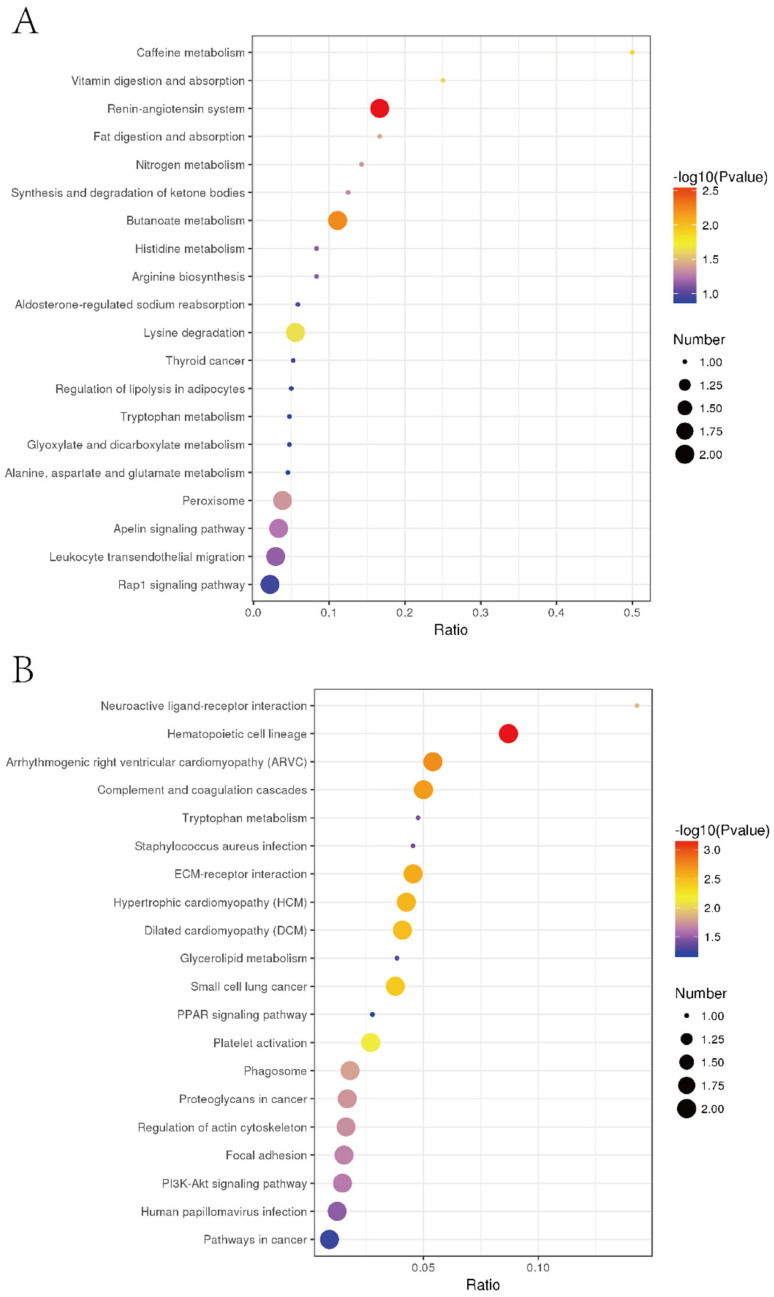
KEGG enrichment results. (**A**) Upregulated; (**B**) downregulated (n = 3). The X-axis is the ratio of the number of DE proteins in the corresponding pathway to the number of total proteins identified in the pathway. The greater the value, the higher the enrichment degree of the DE proteins in the pathway. The colors of the points represent the *p*-value of the hypergeometric test. The redder the color, the smaller the value, indicating that the reliability of the test is greater and more statistically significant. The size of the dot represents the number of DE proteins in the corresponding pathway. The larger the dot, the more DE proteins in the pathway.

To further explore the related pathway of EPSI for anti-hepatoma effects and inhibition of liver tumors, KEGG pathway enrichment analysis was carried out (Figure 8). In the upregulated proteins (Figure 8A), the highest degree of protein enrichment was in caffeine metabolism, while most DE proteins with higher reliability appeared in the renin-angiotensin system. In the downregulated proteins (Figure 8B), two kinds appeared in neuroactive ligand-receptor interaction and hematopoietic cell lineage. The results showed that the inhibition of liver tumors by EPSI was closely related to the above pathways.

In the detailed KEGG pathway enrichment results (Table 5), we found 12 typical enrichment pathways in the process of inhibiting liver tumors by EPSI (*p* < 0.05). The names of the enriched KEGG pathways and the enriched proteins with significant changes are also listed in Table 5, while a description of the proteins is shown in Table 6.

**Figure 9:**
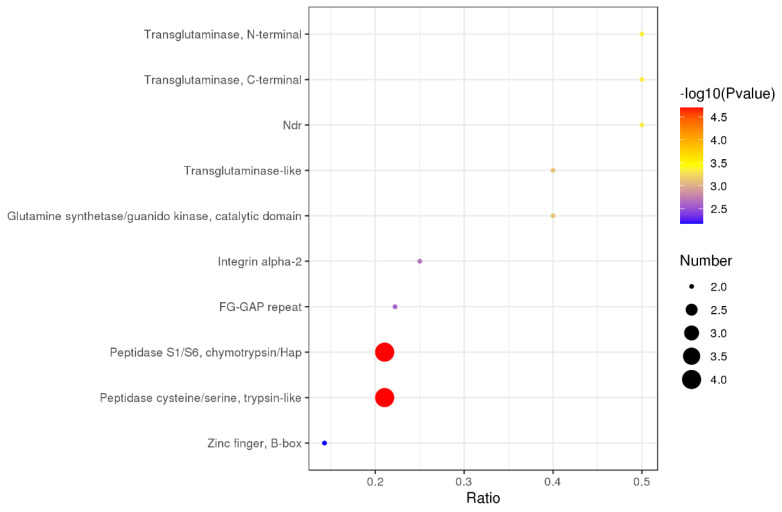
The enrichment results of the domain (n = 3). The X-axis is the ratio of the number of differential proteins in the corresponding domain to the number of total proteins identified in the domain. The greater the value, the higher the enrichment degree of DE proteins in the domain. The colors of the points represent the *p-*value of the hypergeometric test. The redder the color, the smaller the value, indicating that the reliability of the test is greater and more statistically significant. The size of the dot represents the number of DE proteins in the corresponding domain. The larger the dot, the more DE proteins in the domain.

Structural domain enrichment can identify the domain entries that are statistically significantly enriched. This function or positioning may be the cause of the differences. From the statistics of the enrichment results, a bubble chart of the structural domain is shown in Figure 9. As for the previous expression, peptidase S1/S6, chymotrypsin/Hap, peptidase cysteine/serine, and trypsin-like should be considered as the typical structural domains.

### 3.8. Interaction Analysis of Differentially Expressed Proteins

**Figure 10:**
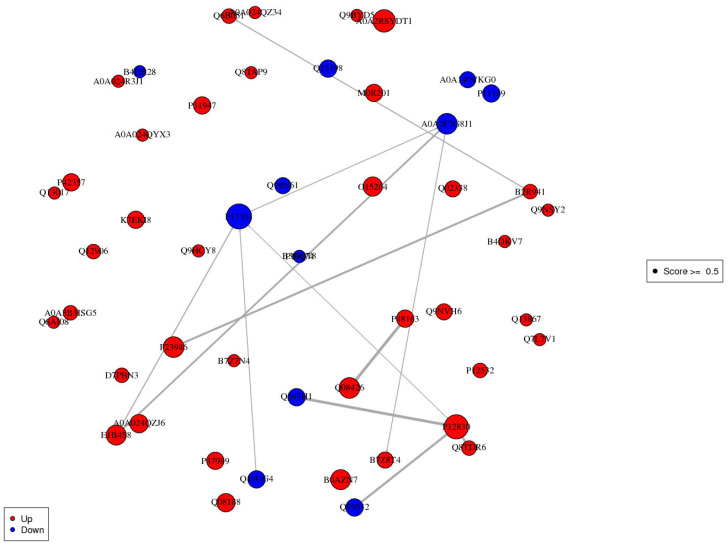
Protein interaction network. Each node in the interaction network represents a protein, and the node size represents the number of interacting proteins. The larger the node is, the more proteins interact with it. The color of the node indicates the expression level of the protein in the comparison pair: red represents significantly high expression of the protein and blue represents significantly low expression of the protein.

According to the KEGG enrichment results (Table 5), we found that a few DE proteins appeared in multiple pathways, such as Q08426, E7ESP4, etc. These results showed that there should be a close relationship between the DE proteins, and between the pathways. The protein interaction network is shown in Figure 10. There are three main associated relationships in the network: the first is centered on P17301, the downregulation of which caused upregulation of other three proteins (E1B4S8, P12830, Q8TDR6) and downregulation of the other four (Q19UG4, A0A0F7G8J1, Q597H1, and Q99542). The second line established the relationships among Q6B051, B2R941, and P23946. In addition, there was also an association between Q08426 and P48163. The above results established the relationship between hematopoietic cell lineage, vitamin digestion and absorption, ARVC, complement and coagulation cascades, and fat digestion and absorption.

However, we were also aware that the situation that the metabolic pathways and related DE proteins summarized above may not be all related to EPSI. Because EPSI is not soluble in water, a certain amount of ethanol must be used as the solvent of EPSI during the feeding of experimental animals. Therefore, the above differences might have the effect of ethanol intervention in the body. We should probably pay more attention to what other pathways the related DE proteins appeared in. Therefore, we summarized the relevant information (Table 7).

In this result, nine DE proteins and 54 related KEGG pathways were shown; meanwhile, the pathways that have been reported to be directly associated with cancer were marked [46,47,48,49,50,51,52,53,54,55,56,57,58,59,60,61,62,63,64,65,66,67,68,69,70,71,72,73,74]. Among these, some are directly related to liver cancer or tumors, such as platelet activation [75], the PI3K-Akt signaling pathway [76], and the PPAR signaling pathway [77], etc. We found that, besides liver cancer, these signaling pathways appeared in studies on cancers of the breast, colon, and thyroid frequently. There are reasons to believe that the inhibitory effect of EPSI on liver tumors may be the same as the inhibitory mechanism of these cancers. We will carry out a demonstration of the metabolic pathways based on the results of this study in follow-up studies.

## 4. Conclusions

In summary, we identified the main components of EPSI by LC-MS and determined that it contained a variety of anti-inflammatory and anti-cancer components. On this foundation, we found that the EPSI showed good growth inhibition of HepG2 cells in vitro, while the high dose of EPSI had an obvious anti-hepatoma effect in vivo, and this effect was better than that of DDP without toxicity. To explore its anti-hepatoma mechanism, a TMT quantitative proteomic approach was used to examine the inhibition of liver tumors by EPSI. DE proteins and their relevance, and the related metabolic pathways were counted and displayed. The upregulated pathways, such as the renin-angiotensin system, and the downregulated pathways, such as hematopoietic cell lineage, and the changes in the PI3K-Akt signaling pathway and the PPAR signaling pathway may be the significant keys to the anti-hepatoma effects. Moreover, the key DE protein intervening in the pathways is probably a new target for the treatment of liver tumors. Moreover, our work shines a new light on the crucial inhibitive function of *S. involucrata* in the progression of liver disease.

## Figures and Tables

**Table 1:** The mobile phase gradient elution process .

Time (min)	Current Speed (μL/mL)	Gradient	B (%)
0–2	300	-	5
2–6	300	Linear	30
6–7	300	-	30
7–12	300	Linear	78
12–14	300	-	78
14–17	300	Linear	95
17–20	300	-	95
20–21	300	Linear	5
21–25	300	-	5

**Table 2:** Peptide fraction separation: liquid chromatography elution gradient table.

Time (min)	Flow Rate (mL/min)	Mobile Phase A (%)	Mobile Phase B (%)
0	1	97	3
10	1	95	5
30	1	80	20
48	1	60	40
50	1	50	50
53	1	30	70
54	1	0	100

**Table 3:** Liquid chromatography elution gradient table.

Time (min)	Flow Rate (nL/min)	Mobile Phase A (%)	Mobile Phase B (%)
0	600	94	6
2	600	85	15
48	600	60	40
50	600	50	50
51	600	45	55
60	600	0	100

**Table 4:** Composition of the extracts from cell suspension cultures of *S. involucrata* (content > 1%).

Name	Formula	CAS	Content (%)
4,5-Dicaffeoylquinic acid	C_25_H_24_O_12_	14534-61-3	29.000
Linolenyl alcohol	C_18_H_32_O	506-44-5	8.453
7-Hydroxycoumarine	C_9_H_6_O_3_	93-35-6	7.691
Chlorogenic acid	C_16_H_18_O_9_	327-97-9	6.134
Metronidazole	C_6_H_9_N_3_O_3_	443-48-1	3.523
Apigenin 7- (6”-crotonylglucoside)	C_25_H_24_O_11_	NA	2.814
9-Oxo-10(E),12(E)-octadecadienoic acid	C_18_H_30_O_3_	54232-58-5	2.618
Cynaroside	C_21_H_20_O_11_	5373-11-5	2.382
Phthalic anhydride	C_8_H_4_O_3_	85-44-9	1.956
Hexadecanamide	C_16_H_33_NO	629-54-9	1.883
3,4,5-tricaffeoylquinic acid	C_34_H_30_O_15_	86632-03-3	1.773
Diisobutyl phthalate	C_16_H_22_O_4_	84-69-5	1.139
Triethyl phosphate	C_6_H_15_O_4_P	78-40-0	1.061

**Table 5:** KEGG enrichment results (top 12, *p* < 0.05).

ID	Title	x	y	n	N	Prot ID
04614	Renin-angiotensin system	2	12	27	3214	B2R941 P23946
00650	Butanoate metabolism	2	18	27	3214	Q02338 Q08426
00380	Tryptophan metabolism	2	21	27	3214	Q9BS61 Q08426
04640	Hematopoietic cell lineage	2	23	27	3214	P17301 E7ESP4
00232	Caffeine metabolism	1	2	27	3214	P47989
04611	Platelet activation	3	74	27	3214	P17301 E7ESP4 O15264
04977	Vitamin digestion and absorption	1	4	27	3214	E1B4S8
00310	Lysine degradation	2	36	27	3214	Q9NVH6 Q08426
03320	PPAR signaling pathway	2	36	27	3214	A0A140VKG0 Q08426
05412	Arrhythmogenic right ventricular cardiomyopathy (ARVC)	2	37	27	3214	P17301 E7ESP4
04610	Complement and coagulation cascades	2	40	27	3214	A0A0F7G8J1 Q19UG4
04975	Fat digestion and absorption	1	6	27	3214	E1B4S8

ID: the ID of the enriched KEGG pathway; Title: the name of enriched KEGG pathway; X: the number of DE proteins associated with this pathway; Y: the number of background (all) proteins associated with the pathway; n: the number of DE proteins annotated by KEGG; N: the number of background (all) proteins annotated by KEGG; Prot ID: the enriched protein list.

**Table 6:** Description of the enriched proteins.

Prot ID	Description
B2R941	cDNA, FLJ94198, highly similar to Homo sapiens carboxypeptidase A3 (mast cell)
P23946	Chymase
Q02338	D-beta-hydroxybutyrate dehydrogenase, mitochondrial
Q08426	Peroxisomal bifunctional enzyme
Q9BS61	Kynurenine 3-monooxygenase
Q08426	Peroxisomal bifunctional enzyme
P17301	Integrin alpha-2
E7ESP4	Integrin alpha-2
P47989	Xanthine dehydrogenase/oxidase
O15264	Mitogen-activated protein kinase 13
E1B4S8	Apolipoprotein B (Fragment)
Q9NVH6	Trimethyllysine dioxygenase
Q08426	Peroxisomal bifunctional enzyme
A0A0F7G8J1	Plasminogen
Q19UG4	Christmas factor (Fragment)

Prot ID: the enriched protein list; description: the function of the protein described in the database.

**Table 7:** KEGG pathways associated with DE proteins which had relationships in the network.

Prot ID	KEGG Pathway
P17301	Hematopoietic cell lineage * Platelet activation * Arrhythmogenic right ventricular cardiomyopathy (ARCV) ECM-receptor interaction * Hypertrophic cardiomyopathy (HCM) Dilated cardiomyopathy (DCM) Small-cell lung cancer * Proteoglycans in cancer * Focal adhesion * Phagosome * Regulation of actin cytoskeleton * PI3K-Akt signaling pathway * Human papillomavirus infection * Pathways in cancer *
E1B4S8	Vitamin digestion and absorption Fat digestion and absorption Cholesterol metabolism *
P12830	Apelin signaling pathway * Thyroid cancer * Rap1 signaling pathway * Bladder cancer * Endometrial cancer * Melanoma * Cell adhesion molecules (CAMs) * Pathogenic Escherichia coli infection * Gastric cancer * Adherens junction * Bacterial invasion of epithelial cells Pathways in cancer * Hippo signaling pathway *
Q19UG4	Complement and coagulation cascades *
A0A0F7G8J1	Complement and coagulation cascades * Neuroactive ligand-receptor interaction * Staphylococcus aureus infection * Influenza A
B2R941	Renin-angiotensin system * Protein digestion and absorption * Pancreatic secretion *
P23946	Renin-angiotensin system *
Q08426	Butanoate metabolism Tryptophan metabolism * Lysine degradation * PPAR signaling pathway * Peroxisome * Beta-alanine metabolism * Propanoate metabolismCarbon metabolism * Metabolic pathways * Fatty acid degradation * Fatty acid metabolism * Valine, leucine, and isoleucine degradation *
P48163	Carbon metabolism * Pyruvate metabolism Metabolic pathways *

Prot ID: the enriched protein list; *: reported to be directly associated with cancer or tumors.

## Data Availability

Raw data can be provided by the corresponding author on request.
